# Genetic Variations and Cisplatin Nephrotoxicity: A Systematic Review

**DOI:** 10.3389/fphar.2018.01111

**Published:** 2018-09-27

**Authors:** Zulfan Zazuli, Susanne Vijverberg, Elise Slob, Geoffrey Liu, Bruce Carleton, Joris Veltman, Paul Baas, Rosalinde Masereeuw, Anke-Hilse Maitland-van der Zee

**Affiliations:** ^1^Department of Respiratory Medicine, Academic Medical Center, University of Amsterdam, Amsterdam, Netherlands; ^2^Department of Pharmacology-Clinical Pharmacy, School of Pharmacy, Bandung Institute of Technology, Bandung, Indonesia; ^3^Division of Medical Oncology and Hematology, Department of Medicine, Princess Margaret Hospital-University Health Network and University of Toronto, Toronto, ON, Canada; ^4^Division of Epidemiology, Dalla Lana School of Public Health, Toronto, ON, Canada; ^5^Division of Translational Therapeutics, Department of Pediatrics, Faculty of Medicine, University of British Columbia, Vancouver, BC, Canada; ^6^BC Children's Hospital Research Institute, University of British Columbia, Vancouver, BC, Canada; ^7^Pharmaceutical Outcomes Programme, British Columbia Children's Hospital, Vancouver, BC, Canada; ^8^Department of Thoracic Oncology, The Netherlands Cancer Institute, Amsterdam, Netherlands; ^9^Division of Pharmacology, Utrecht Institute for Pharmaceutical Sciences, Utrecht, Netherlands

**Keywords:** cisplatin, nephrotoxicity, genetic variations, pharmacogenomics, precision medicine, kidney, toxicity

## Abstract

**Background:** Nephrotoxicity is a notable adverse effect in cisplatin treated patients characterized by tubular injury and/or increased serum creatinine (SCr) with incidence varying from 20 to 70%. Pharmacogenomics has been shown to identify strongly predictive genetic markers to help determine which patients are more likely to experience, for example, a serious adverse drug reaction or receive optimal benefit through enhanced efficacy. Genetic variations have been reported to influence the risk of cisplatin nephrotoxicity; however, a comprehensive overview is lacking.

**Methods:** A systematic review was performed using Pubmed, Embase and Web of Science on clinical studies that used cisplatin-based chemotherapy as treatment, had available genotyping data, and evaluated nephrotoxicity as an outcome. The quality of reporting was assessed using the STrengthening the REporting of Genetic Association Studies (STREGA) checklist.

**Results:** Twenty-eight eligible studies were included; all were candidate gene studies. Over 300 SNPs across 135 genes were studied; 29 SNPs in 14 genes were significantly associated with cisplatin-induced nephrotoxicity. A variation in *SLC22A2* rs316019, a gene involved in platinum uptake by the kidney, was associated with different measures of nephrotoxicity in four independent studies. Further, variants of *ERCC1* (rs11615 and rs3212986) and *ERCC2* (rs13181), two genes involved in DNA repair, were found to be positively associated with increased risks of nephrotoxicity in two independent studies.

**Conclusion:** Three genes consistently associated with cisplatin-induced nephrotoxicity. Further research is needed to assess the biological mechanism and the clinical value of modifying treatment based on *SLCC22A2* and *ERCC1/2* genotypes.

## Introduction

Platinum-based chemotherapeutics, such as cisplatin, carboplatin, and oxaliplatin are among the most widely used antineoplastics for the treatment of solid tumors. Specifically, cisplatin is part of the first-line regimens used to treat head and neck, lung, testis, ovarian, and bladder cancers (Hanigan and Devarajan, 2003; Pabla and Dong, 2008; McWhinney et al., 2009; Wen et al., 2015). Cisplatin [molecular formula: Cl_2_H_6_N_2_Pt; also known as cisplatinum or cis-diamminedichloroplatinum(II)] is a first generation platinum anticancer agent with a square planar geometry metal ion core (Dasari and Tchounwou, 2014). Cisplatin induces cancer cell death by binding to the N7 reactive center of purine residues and causes irreversible DNA damage in cancer cells during division (Dasari and Tchounwou, 2014), thus blocking cell division and promoting apoptosis. Despite its benefit in cancer therapy, cisplatin is also known for its adverse reactions, such as ototoxicity, neurotoxicity, emesis and nephrotoxicity (Percie du Sert et al., 2011; Wensing and Ciarimboli, 2013; Dasari and Tchounwou, 2014).

Cisplatin-induced nephrotoxicity manifests as acute tubular necrosis (Arany and Safirstein, 2003; Hanigan and Devarajan, 2003; Pabla and Dong, 2008; Miller et al., 2010; Stathopoulos, 2013; Derungs, 2015). Since 27–50% of cisplatin is excreted within 48 hours through the kidneys (Gullo et al., 1980), a high concentration of cisplatin and alteration in renal transport mechanisms (Peres and da Cunha, 2013) has been proposed to lead directly to renal inflammation, oxidative damage, apoptosis, and finally to nephrotoxicity (Yao et al., 2007). The efficacy of cisplatin is dose dependent, but the high risk of nephrotoxicity frequently hinders the use of higher doses to maximize its antineoplastic effects (Schellens et al., 2001; Hanigan and Devarajan, 2003). Previous research has demonstrated that high-dose cisplatin can cause severe renal dysfunction in 20% patients (Yao et al., 2007; Peres and da Cunha, 2013), but the incidence may reach as high as 66% in elderly (Peres and da Cunha, 2013) and over 70% in children (Jimenez-Triana et al., 2015). Long-term platinum retention can be found in the plasma of cancer patients even 20 years after discontinuation of cisplatin-based chemotherapy (Gietema et al., 2000; Hjelle et al., 2015), raising concerns about the long-term nephrotoxicity risks over time.

There is a growing interest in the role of genetic variation in the development of cisplatin nephrotoxicity (Liu et al., 2014; Skinner, 2017). Variations in organic transporter molecules genes (Yonezawa and Inui, 2011; Zhang and Zhou, 2012), DNA repair enzyme genes (Khrunin et al., 2010b; Zhang et al., 2012; Xu et al., 2013), tumor suppressor genes (Liu et al., 2014) and metabolic enzymes involved in platinum detoxification (Barahmani et al., 2009; Khrunin et al., 2012) have been associated with the risk of nephrotoxicity. Although several genetic variants have been identified to influence cisplatin-induced nephrotoxicity in oncology patients, a comprehensive overview on which genetic variations are consistently associated with nephrotoxicity-induced cisplatin-based chemotherapy is lacking.

We conducted a systematic review to identify which genetic variants consistently associated with cisplatin-induced nephrotoxicity in oncology patients and assessed whether there are genetic variants that might be clinically relevant to guide cisplatin treatment.

## Methods

We carried out a systematic review according to the Preferred Reporting Items for Systematic Reviews and Meta-Analyses guidelines (PRISMA) (Moher et al., 2009). The protocol was registered in the international prospective register of systematic reviews (PROSPERO; CRD42017064011; Zazuli and Maitland-van der Zee, 2017).

### Data sources and search strategy

Our search strategy included articles indexed in PubMed/MEDLINE, EMBASE and Web of Science. See Supplementary Table 1 for Medical Subject Headings (MeSH) terms and keywords used in this study. Additional research papers were identified by screening the reference sections of included articles.

### Study selection

We constructed a PICOS (population-intervention/exposure-comparison-outcome-study design) framework to set out our review objectives (see Supplementary Table 2). All studies needed to fulfil the following inclusion criteria: (1) genetic association studies, (2) studies using cisplatin-containing chemotherapy, (3) studies that included nephrotoxicity as an adverse outcome (any definition), (4) studies published in the English language, and (5) studies involving cancer patients. Preclinical studies (animal experiment or *in vitro* studies) and studies in which patients were treated with both chemotherapy and radiation therapy were excluded.

After identifying the articles, primary screening by ZZ was performed to determine whether the study met the inclusion criteria based on the abstract. The full paper was evaluated to determine whether an analysis of the association between genetic polymorphisms and platinum induced nephrotoxicity had been performed.

### Data collection and quality assessment

The following data were extracted from each publication: source of study (reference), study design (retrospective, case-control, prospective), setting (type of chemotherapy treatment, type and stage of cancer), patient selection (sample size, inclusion and exclusion criteria), observation period (number of treatment cycles) and nephrotoxicity data (definition, scoring system, level of severity) and genetic polymorphisms (genes investigated, genes involved, name and number of Single Nucleotide Polymorphisms (SNPs), main results).

Two independent reviewers (ZZ and ES) assessed whether the articles met the inclusion criteria. Disagreements were resolved through co-author team discussion. Each reviewer also assessed quality of the reporting of the studies and the risk of bias using a scoring system modified from a previously published study (Leusink et al., 2016) based on STREGA recommendations [see Supplementary Table 3; (Little et al., 2009)]. The scoring system resulted in an overall quality score of 0–10; studies with greater than or equal to half of the maximum points were regarded as of sufficient quality.

### Data analysis

A meta-analysis could not be performed because of substantial differences in outcome definitions where some studies reported categorical outcome variables (e.g., CTCAE and the RIFLE classification [Risk, Injury, Failure, Loss of kidney function, and End-stage kidney disease]) while others reported continuous outcome variables (e.g., differences in serum creatinine [SCr], cystatin C, and estimated glomerular filtration rate [eGFR]). Further, we identified heterogeneity in statistical methods and in the reporting of effect size (few studies reported odds ratios while most reported only *p*-values), or in exposure categorization (i.e., dominant, co-dominant, or additive genetic inheritance models were assumed in different studies). Many articles lacked key data required for meta-analysis, such as the number of subjects experiencing nephrotoxicity per genotype category. Therefore, we report descriptively the results of SNPs that had been found to be associated with nephrotoxicity when they had been assessed in at least two independent study populations.

## Results

### Study eligibility

**Figure 1:**
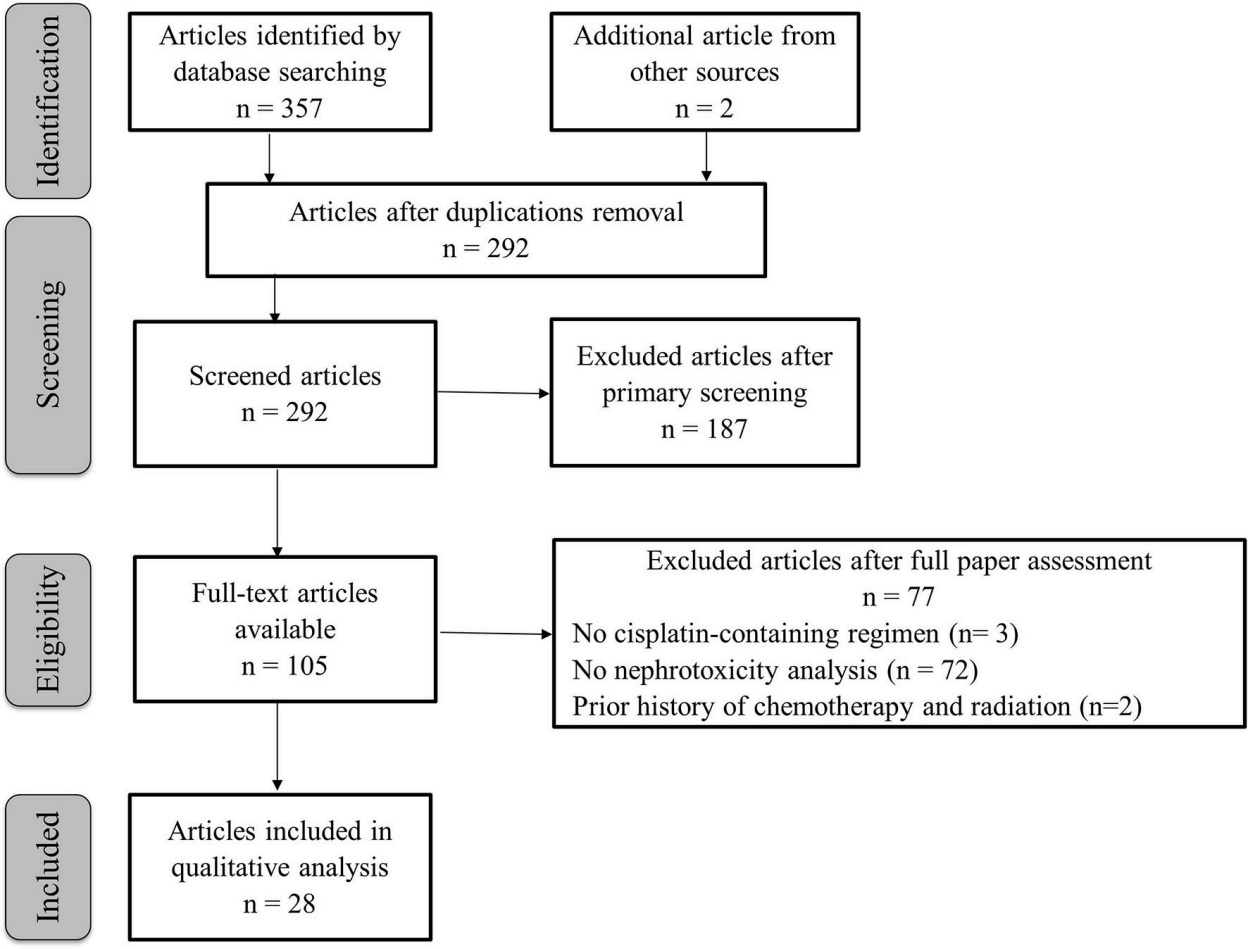
Flow chart of the selection of articles. This flow diagram is created according to the PRISMA recommendation (Moher et al., 2009).

The article selection process is shown in Figure 1. The initial search delivered 359 articles; after removal of duplicates, 292 abstracts were primarily screened of which 105 full-text articles remained. After reading the full-text, 77 publications were excluded: three studies did not investigate cisplatin-based chemotherapy regimens, 72 studies did not evaluate relationships with nephrotoxicity, and two studies were confounded by concurrent radiation treatment. In the end, 28 studies were analyzed.

### Study characteristics

The study characteristics of the 28 articles included, all were candidate gene studies published between 2008 and 2017, are shown in Table 1. Most were cohort studies (Wang et al., 2008; Filipski et al., 2009; Goekkurt et al., 2009; Chen et al., 2010; Khrunin et al., 2010a, 2012, 2014; KimCurran et al., 2011; Tzvetkov et al., 2011; Erculj et al., 2012; Iwata et al., 2012; Windsor et al., 2012; Xu et al., 2012, 2013; Zhang and Zhou, 2012; Zhang et al., 2012; Hinai et al., 2013; Khokhrin et al., 2013; Lamba et al., 2014; Yuan et al., 2015; Hattinger et al., 2016; Powrozek et al., 2016; Chang et al., 2017) and none were genome wide association studies (GWAS). Key details of subject characteristics [e.g., ethnicity and inclusion criteria (Sprowl et al., 2012)], type of chemotherapy regimens (Erculj et al., 2012; Sprowl et al., 2012; Powrozek et al., 2016), and nephrotoxicity criteria (Goekkurt et al., 2009; Kim et al., 2012; Khrunin et al., 2014) were not reported in some studies.

**Table 1:** Included pharmacogenetic studies of cisplatin-induced nephrotoxicity.

**Study, year of publication, country (References)**	**Subjects**	**SNPs investigated**	**Chemotherapy regimen**	**Definition of nephrotoxicity**	**Results**
Wang et al., 2008, China	*n* = 139 Han Chinese, Median age: 55 years (21–73); advanced NSCLC and extensive SCLC	XRCC1 rs1799782 XRCC1 rs25487	Vinorelbine + cisplatin Paclitaxel + cisplatin Docetaxel + cisplatin Gemcitabine + cisplatin Etoposide + cisplatin	NCI-CTCAE v3.0 grade 0–2 vs. 3–4	There were no grades 3 and 4 renal toxic effects.
Filipski et al., 2009, The Netherlands	Not specified in detail. Subjects with malignant solid tumors.	OCT2 808G > T (rs316019)	Cisplatin-based regimens	Changes in serum creatinine after the 1st cycle	Subjects carrying a copy of this SNP (*n* = 10; 13%) experienced no change in serum creatinine after cisplatin treatment (*p* = 0.12), whereas serum creatinine significantly increased in patients carrying the wild-type (*n* = 68; *p* = 0.0009).
Goekkurt et al., 2009, Germany	*n* = 134 (FLO = 71, FLP = 63) Subjects genotyped = 133. Age: 64 years (27–86) Advanced gastric cancer (AGC) with various stage	1. XPD rs13181 2. XPD rs1799793 3. XRCC1 rs25487 4. XPA rs1800975 5. ERCC1 rs11615 6. ERCC1rs3212986 7. GSTM1 (deletion) 8. GSTT1 (deletion) 9. GSTP1 rs1695 10. TS-1494del6 11. TS-VNTR (28bp repeat) 12. TS-VNTR + G/C SNP] 13. MTHFR rs1801133 14. MTHFR rs1801131 15. MTR rs1805087 16. OPRT rs1801019	FLO (fluorouracil-leucovorin-oxaliplatin) and FLP (fluorouracil-leucovorin-cisplatin)	Not defined; using grading system from 0 to 4. Grouped into grade 0–2 vs. 3–4.	Significant associations between the XPD-Asn312-751Gln (rs1799793-rs13181) haplotype and nephrotoxicity. OR = 2.27 (95% CI 1.28–4.0, *P* = 0.005)
Khrunin et al., 2010b, Russia	*n* = 104 Russian women from eastern Slavonic origin with epithelial ovarian cancer (stages I–IV)	1. GSTA1 rs3957357 2. GSTM1 Gene deletion 3. GSTM3 3-bp deletion, rs1799735 4. GSTM3 rs7483 5. GSTP1 rs1695 6. GSTP1 rs1138272 7. GSTT1 Gene deletion 8. ERCC1 rs11615 9. ERCC1 rs3212986 10. XPD rs1799793 11.XPD rs13181 12. XRCC1 rs1799782 13. XRCC1 rs25489 14. XRCC1 rs25487	Cisplatin + cyclophosphamide	NCI-CTCAE (version not mentioned). Grouped into grade 0 vs. ≥1.	Cases of renal dysfunction were more prevalent among patients with the ERCC1 gene rs11615 heterozygous T/C (46.7%) with OR = 2.51 (95% CI 1.09–5.57; *p* = 0.037) and rs3212986 heterozygous C/A (52.8%) genotypes with OR = 3.29 (95% CI 1.40–7.73; *p* = 0.009) compared with the homozygous variants.
		15. TP53 16-bp duplication rs17878362 16. TP53 rs1042522 17. TP53 rs1625895 18. CYP2E1 96-bp insertion 19. CYP2E1 rs2031920 20. CYP2E1 rs6413432 21. CYP2E1 rs2070676			
Khrunin et al., 2010a, Russia	*n* = 104 Russian women East Slavonic origin, median age 52 years (23–65 years)	1. DNASE1 (intron 4) VNTR 2. DNASE1 (exon 8) SNP, rs1053874 G/A, Arg244, Gln 3. GGT1 (5′ region) SNP, rs2236626 C/T 4. GGT1 (intron 1) SNP, rs5751901 C/T 5. OCT2 (exon 4) SNP, rs316019 G/T, Ala244, Ser 6. HO1 (5′ region) SNP, rs2071746 A/T	Cisplatin + cyclophosphamide	standard WHO criteria (Nephrotoxicity assessed as a decrease in creatinine clearance below 60 ml/min)	An increased risk of nephrotoxicity was noted for patients with the homozygous GGT1 T/T genotype (rs5751901)
Chen et al., 2010, China	*n* = 95 Age median: 58 (35–77), Chinese, advanced NSCLC (stages IIIB-IV)	1. ERCC1 rs11615 2. MDR1 [E1/-129(T/C)] 3. MDR1 rs2032582 4. MDR1 rs1045642	Cisplatin + gemcitabine Cisplatin + vinorelbine Cisplatin + taxane	WHO toxicity criteria (1979); grouped into grade 0 vs. ≥1.	No significant association between the MDR1 gene rs2032582, E1/-129(T/C), rs1045642 or ERCC1 gene rs11615 polymorphisms and the risk of hematologic toxicity, gastrointestinal toxicity, hepatotoxicity, or nephrotoxicity (*P* > 0.05).
Tzvetkov et al., 2011, Germany	*n* = 79 Caucasian. age 58 years (22–76) Type of cancer: Lung cancer (55.6%), 14 had esophageal or stomach cancer (17.3%), six had non-Hodgkin's lymphoma (7.4%), and 16 had other cancers (19.8%); all at late-stage cancer	1. GSTM1 Whole-gene deletion 2. LARP2, rs4834232 and rs6848982 3. OCT1, Number of active alleles 4. OCT2, rs316019 5. EPO, rs1617640 6. Intergenic, rs7131224 7. XRCC1, rs25489 and rs1799782 8. ERCC1, rs3212986 and rs11615 9. GSTT1, Whole-gene deletion	Cisplatin-containing chemotherapy.	Relative change in the estimated glomerular filtration rate (eGFR), serum creatinine and cystatin C levels.	Homozygous carriers of the rare A allele of the 8092C > A ERCC1 gene polymorphism (rs3212986) were protected from cisplatin-induced nephrotoxicity (mean change in eGFR: AA = 24 ± 3.4 ml/min/1.73m2); CA = −10.2 ± 2.6 ml/min/1.73m2; CC = −12.6 ± 2.5 ml/min/1.73m2, *P* = 0.0002). Homozygous carriers of the C allele of the Asn118Asn ERCC1 gene polymorphism were also protected from cisplatin-induced nephrotoxicity (mean change in eGFR: CC = 6.91 ± 9.1 ml/min/1.73m2; CT = −11.8 ± 1.7 ml/min/1.73m2; TT = −12.8 ± 4.2 ml/min/1.73m2, *P* = 0.004)
Erculj et al., 2012, Slovenia	*n* = 113 Median age: 60 (32–83)	1. XPD rs1799793 2. XPD rs13181	Various platinum-based regimens	NCI-CTCAE v2.0 Grade 0 vs. Grade 1–4	None of the investigated polymorphisms influenced the occurrence of nephrotoxicity.
	Malignant mesothelioma (various stage)	3. ERCC1 rs11615 4. ERCC1 rs3212986 5. GSTP1 rs1695 6. GSTP1 Ala114Val 7. GSTM1 CNV 8. GSTT1 CNV			
KimCurran et al., 2011, China	*n* = 300 Chinese, age median = 60 years (33–78) Stage IIIB/IV NSCLC	ERCC1 rs3212986	Platinum + gemcitabine Platinum + vinorelbine Platinum + taxane	NCI-CTCAE v3.0 Grade 0 vs. Grade 1–4	No significant association between polymorphism and nephrotoxicity (*p* = 0.311)
Xu et al., 2012, China	*n* = 282 Han Chinese, age (median) 56 years (34–76) Type of cancer NSCLC, stage IIIA-IV	22 SNPs of eIF3a gene	cisplatin/carboplatin + gemcitabine; cisplatin/carboplatin + etoposide and cisplatin + docetaxel; cisplatin/carboplatin + gemcitabine; cisplatin/carboplatin + etoposide; cisplatin + docetaxel	NCI-CTCAE v3.0 moderate = grade 0 vs. severe = grade 1–4	Minor T-carrier of eIF3a Arg803Lys C > T polymorphism have better tolerance than C-carrier to cisplatin-induced toxicity in NSCLC patients (moderate = 42, 14.89% vs. severe = 34, 12.06%; *P* = 0.01) overall OR cisplatin + carboplatin for T-carrier (T alelle) = 0.54 (95% CI 0.32–0.93, *P* = 0.03)
Kim et al., 2012, South Korea	*n* = 129 Korean, age median: 63 years (38–78) Type of cancer: unresectable NSCLC stage III-IV	ERCC2 gene: rs13181 rs1799787 rs238405 rs238415 rs238416 rs3916874 rs50871 rs50872	Weekly schedule of cisplatin or carboplatin; Biweekly cisplation; or a 3-week schedule of cisplatin or carboplatin plus either gemcitabine or taxane (weekly schedule of docetaxel; biweekly schedule of docetaxel; a 3-week schedule of docetaxel; a weekly schedule of paclitaxel) for stage IIIA or IIIB patients treated with concurrent chemoradia- tion; or a 3-week schedule of paclitaxel	Not defined; using grading system from 0 to 4. Grouped into grade 0–2 vs. 3–4.	rs238405 was significantly associated with infection and nephrotoxicity (*p* < 0.05). In addition, there were significant associations between rs238415 and nephrotoxicity (*p* < 0.05), rs238416 and nephrotoxicity and asthenia (*p* < 0.05).
Windsor et al., 2012, UK	*n* = 50 Patients aged >16 years who had completed MAP chemotherapy for histologically proven osteosarcoma.	Folate pathway 1. MTHFR rs1801133, 1801131, 4846051, 2274976 2. MTHFD1 rs1950902, 2236225 3. RFC rs1051266 4. DHFR 5q11.2 5. TS 18p11.32 ABC efflux 6. ABCB1 (MDR1) rs1128503, 1045642 7. ABCG2 (BCRP) rs2231142 8. ABCC1 (MRP1) rs246240, 3784862	MAP (methotrexate, adriamycin (doxorubicin), cisplatin)	NCI-CTCAE v3.0 Grouped into grade 0 vs. grade 1–4	The regimens may cause decreased of GFR. SNPs below involved in early toxicity: 1. ERCC2 c.2251A > Cp.Lys751Gln (rs13181). AC/CC vs. AA; OR = 4.4 (95% CI 1–18.8, *P* = 0.044); decrease of GFR = 23 vs. 4 mL/min/1.73 m2, *p* < 0.05. 2. MTHFR c.677C > T p. Ala222Val rs1801133 (sex as additional covariate). CT/TT vs. CC = 33 vs. 20.5 mL/min/1.73 m2, *p* < 0.05
		9. ABCC2, rs717620, 2273697, 17222723, 8087710			
		DNA repair 10. ERCC1 rs3212986, 11615 11. ERCC2 rs13181 12. ERCC4 rs1800067 13. XRCC3 rs861539 14. XPC rs2228001 GST enzymes 15. GSTP1 rs1695, 1128272 16. GSTT1 22q11.23 17. GSTM1 1p13.3 Others 18. CBR3 rs8133052, 1056892 Gene 19. CCND1 rs9344 20. NQO1 rs1131341, 1800566 21. NADPH NCF4 rs1883112 22. CYBA rs4673			
Zhang, et al. 2012, Japan	*n* = 365 Asian, Age median 60 years (30–78) Type of cancer: NSCLC (IIIB and IV)	ERCC5 rs17655 ERCC6 rs2228526; Q1413R, A/G; R1213G, A/G CCNH rs2266690 MMS19L, G811A, G/A XPC, Q940K, A/C; R500W, C/T RRM1 rs12806698	vinorelbine or gemcitabine, or docetaxel or paclitaxel plus cisplatin or carboplatin	NCI-CTCAE v3.0 Grouped into grade 0 vs. 1–4.	SNPs of MMS19L G811A may have some roles in predicting the increase of creatinine during chemotherapy in NSCLC OR = 4.436 (95% CI 2.018–10.372, *P* = 0.013)
Iwata et al., 2012, Japan	*n* = 53 Age 68.0 ± 9.7 years Type of cancer: various advanced carcinomas	OCT2 rs316019 MATE1 rs2289669G > A	Cisplatin. The number of patients who were administered drugs in combination was gemcitabine (15),5-fluorouracil (14), etoposide (7), docetaxel and 5-fluorouracil (6), vinorelbine (6), pemetrexed (4), and irinotecan (1).	NCI-CTCAE v4.0 Grouped into grade 0–1 vs. ≥grade 2	The 808G/T SNP in OCT2 (rs316019) ameliorated CDDP-induced nephrotoxicity without alteration of disposition (decrease of SCr on wild type GG vs. GT = 1.11 ± 0.37 vs. 0.92 ± 0.15, *P* = 0.04) but not significant on other parameters e.g. eGFR and BUN, whereas the rs2289669 G/A SNP in MATE1 had no effect on CDDP toxicity. GT vs. GG, *P* = 0.08
Zhang et al., 2012, China	*n* = 123 Age 54.62 ± 10.35 years (18–75) Han Chinese, malignant solid tumor any stage	SLC22A2 808 G > T (rs316019)	Cisplatin alone or in combination with docetaxel and etoposide.	Changes in biomarkers of renal function, i.e. serum creatinine (SCr), blood urea nitrogen (BUN) and cystatin C.	No significant association between the increased of SCr to the genetic variations (GG 0.833 ± 7.394 vs. GT/TT 2.091 ± 6.302 mmol/L; *P* = 0.346) but there is a significant association between Cystatin C and genetic variations (GG 0.043 ± 0.107 vs. GT/TT −0.013 ± 0.120 mmol/L; *P* = 0.009)
Sprowl et al., 2012, country not mentioned	*n* = 112 White patient; Type of cancer and its staging was not defined	ABCC2: rs1885301 rs2804402	Various cisplatin based regimens	changes in serum creatinine levels following treatment	Changes in serum creatinine levels were not significantly linked with any of the common genotypes.
		rs17216177 rs717620 rs2273697 rs3740066 4544G > A			
Khrunin et al., 2012, Russia	*n* = 87 Age < 65 years (median = 51) Yakut women with no history of interethnic marriages in the past two generations Type of cancer: epithelial ovarian cancer, staging from I to IV	1. GSTA1 rs3957357 2. GSTM1 gene deletion 3. GSTM3 AGG/deletion 4. GSTM3 rs7483 5. GSTP1 rs1695 6. GSTP1 rs1138272 7. GSTT1 gene deletion 8. ERCC1 rs11615 9. ERCC1 rs3212986 10. ERCC2 rs1799793 11. ERCC2 rs13181 12. XRCC1 Arg194Trp 13. XRCC1 rs25489 14. XRCC1 rs25487 15. TP53 rs1042522 16. CYP2E1 96-bp insertion 17. CYP2E1-1053C > T 18. CYP2E1 rs6413432 19. CYP2E1 rs2070676	cisplatin + cyclophosphamide	NCI-CTCAE v2.0 Tolerable:grade 0 vs. Severe: grade 1–4	Yakut patients with the GSTT1-null genotype had a higher risk for nephrotoxicity with OR = 3.31 (95% CI 1.15–9.54; *p* = 0.028)
Hinai et al., 2013, Japan	*n* = 95 Age: 65.8 ± 7.7 (51–89) Oesophageal cancer, Stage II-IV	SLC22A2 rs316019	Cisplatin + fluorouracil	NCI-CTCAE v4.0	Cisplatin-induced nephrotoxicity seems to be unaffected by SLC22A2 808G > T polymorphism. (SCr difference: GG −0.30 ± 0.30, GT/TT −0.40 ± 0.53, *p* = 0.25)
Khokhrin et al., 2013, Russia	*n* = 87 Yakut women with ovarian cancer (stage I-IV)	228 polymorphic loci of 106 genes	Cisplatin + cyclophosphamide	NCI-CTCAE v2.0	No result regarding nephrotoxicity reported. Probably its not significantly associated.
Khrunin et al., 2014, Russia	*n* = 104 median age 52 years (23–65) Russian women from eastern Slavonic origin with epithelial ovarian cancer (stages I-IV)	228 SNPs in 106 genes	cisplatin + cyclophosphamide	Standard criteria (not mentioned). Grouped into grade 0 vs. 1–4.	Nephrotoxicity was more frequent among patients with a heterozygous genotype CT of EPHX gene rs1051740 (OR = 9.524, 95% CI 3.621–225.520, *p* = 0.000003). Patients with toxicity: CT vs. TT vs. CC = 25/39 vs. 9/57 vs. 1/6.
Liu et al., 2014, Taiwan	*n* = 116 Age: case 63.43 ± 9.47, control 66.66 ± 11.64 Type of cancer and its staging was not defined	1. TP53 rs1042522 2. ERCC1 rs11615	Platinum + gemcitabine Platinum + etoposide Platinum + vinorelbine Platinum + taxane	RIFLE category	No significant correlation between genetic variations and nephrotoxicity
Lamba et al., 2014, USA	*n* = 90 (only 2 subjects receive cisplatin) White patients, stage IIIB/IV NSCLC	NQO1 rs1800566 TMEM63A rs10158985 ABCC1 rs246240 ABCC1 rs2238476	87 received carboplatin, two received cisplatin, and two received cisplatin and carboplatin as first-line chemotherapy.	NCI-CTCAE v4.0 Grade 0–2 vs. grade 3–4	No toxicity experience by subjects
		ABCB1 rs1128503 KCNC1 rs17718902 CCDC127 rs9312960			
Powrozek et al., 2016, Poland	*n* = 55 Age 64 ± 7 years (51–77) Caucasian, inoperable, locally advanced or advanced NSCLC (IIIB and IV)	ERCC1 rs3212986 ERCC1 rs11615 XPD/ERCC2 rs13181 XPD/ERCC2 rs1799793 XPC rs2228001 XPC rs2228000 RRM1 (−37C > A) RRM1 rs11030918 XPA (−4A > G) XRCC1 rs25487 XRCC1 rs1799782 STMN1 rs182455 XPG/ERCC5 (3310C > G)	platinum compounds + vinorelbine	NCI-CTCAE v4.03 Severe: grade 2–4	Risk of early severe nephrotoxicity (after 2nd cycle) was significantly lower in carriers of C allele of XPD gene (rs13181, 2251A > C, OR = 0.07, 95% CI 0.02–0.31, *P* < 0.0001) than in patients with A allele of this gene. Risk of severe nephrotoxicity after 4th chemotherapy cycle was significantly lower in carriers of C allele (rs13181, 2251A > C, OR = 0.24, 95% CI 0.07–0.81, *P* = 0.017) and A allele (rs1799793, 934G > A, OR = 0.26, 95% CI 0.07–0.90, *P* = 0.029) of XPD gene compared to patients carried A or G allele of this gene
Yuan et al., 2015, China	*n* = 47 Chinese (aged 29–74 years, with the median age of 59); NSCLC stage III-IV	GSTP1 rs1695 RRM1 C37A-T524C (rs 12806698-rs 11030918)	gemcitabine-cisplatin	1979 WHO criteria (acute and subacute toxicity graduation criteria in chemotherapeutic agents).	No participant developed nephrotoxicity.
Hattinger et al., 2016, Italy	*n* = 57 Caucasian, age: median 64 ± 7 (51–77), high-grade osteosarcoma (HGOS)	45 polymorphisms and two haplotypes of 31 genes involved in transport, metabolism, activation and detoxification of the four drugs used in standard HGOS chemotherapy (methotrexate, doxorubicin, cisplatin and ifosfamide)	doxorubicin, high-dose methotrexate, cisplatin and ifosfamide	NCI-CTCAE v3.0 (graded from 0 to 4)	Nephrotoxicity and stomatitis were registered in one patient each (2%) and were therefore not included in association analyses.
van der Schoot et al., 2016, The Netherlands	*n* = 369 Age median = 28 years (16–64); metastatic testicular cancer	HFE: rs1799945 rs1800562	Bleomycin + cisplatin	Serum concentration creatinine (mmol/l), and calculated CRCL (Cockcroft-Gault formula).	Renal function, by means of serum creatinine level and calculated CRCL before chemotherapy, 6 weeks after the last course of chemotherapy, 1 year after start of chemotherapy, and 10 years after start of chemotherapy, was similar in patients with or without HFE gene variants
Chang et al., 2017, USA	*n* = 206 Age: 53 ± 14 years; various type of cancer	Selected polymorphisms of interest including transporters (SLC22A2, ABCC2, SLC47A1), regulatory (NFE2L2, KEAP1) and metabolism (GSTA1, GSTP1, GGT1) genes	Various cisplatin-based combination (etoposide, vinblastine, dacarbazine, aldesleukin, interferon alfa 2b, gemcitabine, docetaxel)	Changes in protein biomarkers (e.g., calbindin, clusterin, KIM-1, GST-pi, IL-18, MCP-1, albumin, B2M, cystatin C, NGAL, osteopontin, TFF3)	The polymorphisms rs596881 (SLC22A2/OCT2), and rs12686377 and rs7851395 (SLC31A1/CTR1) were associated with renoprotection and maintenance of estimated glomerular filtration rate (eGFR). Polymorphisms in *SLC22A2*/OCT2, *SLC31A1*/CTRI, *SLC47A1*/MATE1, *ABCC2*/MRP2, and *GSTP1* were significantly associated with increases in the urinary excretion of novel AKI biomarkers: KIM-1, TFF3, MCP1, NGAL, clusterin, cystatin C, and calbindin.

### Study population

Of 3,799 adult subjects across these 28 studies, 1,443 patients (*n* = 13 studies) were predominantly of European Caucasians and 1,948 (*n* = 12 studies) were performed in East Asian populations. However, most studies did not explain how ancestry was determined. Individual study sizes ranged from 47 to 365 patients. Only six studies included more than 200 patients, while 12 studies had fewer than 100 subjects.

Ten studies involved lung cancer patients; five involved ovarian cancer; one study each evaluated gastric cancer, osteosarcoma, esophageal cancer, testicular cancer, and mesothelioma patients; six studies included various cancer types or did not mention the cancer type.

### Chemotherapy regimens used

Different cancer sites used diverse drug combinations with cisplatin, and most studies evaluated more than one type of cisplatin combination therapy. Combinations with other drugs (Moon et al., 2011) and the dosage of cisplatin (Bennis et al., 2014) can influence the incidence of nephrotoxicity. Cisplatin + gemcitabine was the most commonly reported treatment regimen (*n* = 11 studies), followed by cisplatin + taxane (either cisplatin + docetaxel or cisplatin + paclitaxel, *n* = 10), cisplatin + etoposide (*n* = 7), cisplatin + cyclophosphamide (*n* = 5) and cisplatin + vinorelbine (*n* = 5). Cisplatin dosage also varied widely among studies: the lowest dose mentioned was 20 mg/m^2^ and the highest was 100 mg/m^2^ under a three-weekly schedule.

### Outcome

The National Cancer Institute Common Terminology Criteria of Adverse Events (NCI-CTCAE) (Institute, 2016) criteria was the most commonly used classification of drug-induced nephrotoxicity (*n* = 15). Serum creatinine was used for classifying the severity of nephrotoxicity. The NCI-CTCAE 4.03 grading of acute kidney injury were as follows (Institute, 2016): Grade 1, a creatinine level increase of >0.3 mg/dL or a creatinine level that was 1.5–2.0 times above baseline; Grade 2, a creatinine level that was 2–3 times above baseline; Grade 3, either a creatinine that was >3 times above baseline or an absolute creatinine level of over 4.0 mg/dL or any rise that required hospitalization; Grade 4, life-threatening consequences or dialysis indicated. However, we found marked variability in the standard grading to determine nephrotoxicity. Eight studies defined nephrotoxicity as ≥grade 1, while three studies described nephrotoxicity as ≥grade 2, two studies provided no information regarding the standard, and other studies used changes in creatinine serum (*n* = 4), WHO criteria (*n* = 3), changes in creatinine clearance (*n* = 2) or changes in a novel urinary biomarker (*n* = 1). Three studies did not describe the nephrotoxicity criteria at all.

### Quality assessment and quality of reporting

Quality assessment was performed by two reviewers (ZZ and ES) using recommendations for reporting genetic association studies [(Little et al., 2009); Supplementary Table 4]. Only 16 studies (42.8%) provided sufficient information according to the STrengthening the REporting of Genetic Association Studies (STREGA) recommendations. One of the items in the predetermined reporting criteria (multiple testing correction) was not reported by most studies because unlike GWAS, the majority of the studies were not investigating multiple genetic markers at once. Therefore, a large number of studies (*n* = 22; 79%) received a lower quality score. Note that if certain reporting requirements are of low relevance to the circumstances of the individual article, lower quality reporting scores do not necessarily reflect the research quality. As many as 24 studies (92%) did not report the power and sample size calculation, of which 12 studies (43%) showed no statistically significant associations. Nevertheless, seven of eight studies that showed a statistically significant association for *ERCC1, ERCC2* and *SLC22A2* also did not report power or sample calculations. Nine of 14 studies that showed no statistically significant association failed to report whether there were issues concerning genotyping quality, for example, by reporting the percentage of successful genotyping attempts or a cross validation with a different genotyping technique. For nine studies (32%), there were concerns about the quality of the study design and analysis. For example, the authors did not calculate and interpret the statistical interaction adequately. Within their Results sections, 15 and 10 studies did not report participants' characteristics and outcomes stratified by genotype, respectively. As many as 13 studies did not mention limitations of the study and sources of potential bias. In 19 studies (67.9%), the clinical information was of sufficient quality as the authors mentioned the specific cisplatin-based chemotherapy regimens and the dosage per cycle and the number of cycles, and there was an adequate methodologic description including participant selection, baseline characteristics, inclusion criteria, nephrotoxicity criteria (including objective lab parameters).

### Genes studied in cisplatin-induced nephrotoxicity

Candidate genes of all 28 studies were chosen based on platinum pharmacokinetic or pharmacodynamic pathways. As many as 135 genes involved in DNA repair, drug transport, tumor suppression, regulation of intracellular process, or detoxification were investigated. The number of variants assessed per study ranged from 1 to 228 SNPs. From those 135 genes, 14 genes were associated with cisplatin-induced nephrotoxicity in at least one study: *ERCC1* and *ERCC2* (XPD), *SLC22A2* (OCT2), *SLC31A1* (CTR1), *SLC47A1* (multidrug and toxin extrusion protein 1; MATE1), *ABCC2* (multidrug resistance protein 2; MRP2), Kelch-like ECH-associated protein 1 (*KEAP1*), nuclear factor erythroid derived 2 like 2 (*NFE2L2*), *GSTP1* (Glutathione S-Transferase Pi 1), *GSTT1*, methylene tetrahydrofolate reductase (*MTHFR)*, epoxide hydrolase 1 (*EPHX1)*, eukaryotic translation initiation factor 3 subunit A (*eIF3a)* and *MMS19L*. However, only SNPs from *ERCC1, ERCC2* (XPD), and *SLC22A2* (OCT2), consistently showed a positive association in at least two studies (Table 2).

**Table 2:** Effect size of SNPs that had been replicated at least one positive association.

**Outcome**	**Study**	**Value in variants genotype/allele**	**Value in reference genotype/allele**	**OR (95% CI)**	**Mean difference**	***P***
***ERCC1 rs11615***						
CTCAE >grade 0	Khrunin et al., 2010b, Russia	CT (46.7%)	TT/CC (no values reported)	2.51 (1.09–5.57)	N/A	0.037
Change in the estimated glomerular filtration rate (eGFR)	Tzvetkov et al., 2011, Germany	CC = 6.91 ± 9.1 ml/min/1.73 m^2^	TT/CT CT = −11.8 ± 1.7 ml/min/1.73 m^2^ TT = −12.8 ± 4.2 ml/min/1.73 m^2^	N/A	N/R	0.004
***ERCC1 rs3212986***						
CTCAE >grade 0	Khrunin et al., 2010b, Russia	CA (52.8%)	CC/AA (no values reported)	3.29 (1.40–7.73)	N/A	0.009
Change in the estimated glomerular filtration rate (eGFR)	Tzvetkov et al., 2011, Germany	AA = 24 ± 3.4 ml/min/1.73 m^2^	CC/CA CA = −10.2 ± 2.6 ml/min/1.73 m^2^ CC = −12.6 ± 2.5 ml/min/1.73 m^2^	N/A	N/R	0.0002
***ERCC2 rs13181***						
Change in eGFR	Windsor et al., 2012, the UK	AC/CC = −23 mL/min/1.73 m^2^	AA = −4 mL/min/1.73 m^2^	N/A	−19 mL/min/1.73 m^2^	0.021
CTCAE >grade 0	Windsor et al., 2012, the UK	AC/CC (no values reported)	AA (no values reported)	4.4 (1–18.8)	N/A	0.044
CTCAE >grade 1	Powrozek et al., 2016, Poland	C allele (no values reported)	A allele (no values reported)	0.07 (0.02–0.31)	N/A	< 0.0001
***SLC22A2 rs316019***						
% changes in serum creatinine after the 1st cycle	Filipski et al., 2009, Netherlands	GT (no values reported)	GG (no values reported)	N/A	N/R	0.0009
Increase of SCr	Iwata et al., 2012, Japan	GT = 0.92 ± 0.15 mg/dL	GG = 1.11 ± 0.37 mg/dL	N/A	−0.19 mg/dL	0.04
	Zhang et al., 2012, China	GT/TT = 2.091 ± 6.302 mg/dL	GG = 0.833 ± 7.394 mg/dL	N/A	1.258 mg/dL	0.346
Changes in cystatin C.	Zhang et al., 2012, China	GT/TT = −0.013 ± 0.120 mmol/L	GG = 0.043 ± 0.107 mmol/L	N/A	0.056 mmol/L	0.009
Fold changes in protein biomarkers (KIM-1)	Chang et al., 2017, the USA	GT (no values reported)	GG (no values reported)	N/A	1.77 × 10^171^	0.038

*SNPs, single nucleotide polymorphisms; OR, odds ratio; CTCAE, Common Terminology Criteria for Adverse Effects; SCr, serum creatinine; KIM-1, kidney injury molecule-1; N/A, not available; N/R, not reported*.

### Genetic polymorphisms in ERCC1

Polymorphisms in the nucleotide excision repair genes *ERCC1* and *ERCC2* (XPD) have been linked to alterations of the DNA repair process and capacity (Giachino et al., 2007; Friboulet et al., 2013; Xiong et al., 2017); this is postulated to affect nephron repair after injury by platinum agent exposure. Furthermore, *ERCC1* may affect target cell sensitivity to platinum-based therapy (Li et al., 2012, 2014; Bogush et al., 2015; Han et al., 2016) and patient response (Ryu et al., 2004; Cheng et al., 2012; Lv et al., 2014; Kaewbubpa et al., 2016; Li et al., 2016). Carriers of the wild-type C/C genotype of *ERCC1* C118T (CC) (rs11615) had a higher chance of responding to platinum-based chemotherapy than patients carrying variant alleles (Cheng et al., 2012; Lv et al., 2014).

SNPs of *ERCC1* (synonymous rs11615 and rs3212986 located at the 3′ UTR) were the most studied polymorphisms for cisplatin-induced nephrotoxicity. Associations between this gene and cisplatin nephrotoxicity were reported in two studies by (Khrunin et al., 2010b) and (Tzvetkov et al., 2011) in which the same SNPs (rs11615 and rs3212986) were investigated. (Khrunin et al., 2010b) observed that an increased risk for renal dysfunction was observed among epithelial ovarian cancer patients carrying the heterozygous genotype (TC) of rs11615 (46.7%) with an OR = 2.51 (95% CI 1.09–5.57; *P* = 0.037) and for carriers of the rs3212986 CA genotype (52.8%) with an OR = 3.29 (95% CI 1.40–7.73; *P* = 0.009), when compared with the patients carrying the homozygous variant genotype. (Tzvetkov et al., 2011) confirmed these results reporting that both SNP variants were statistically significantly associated with a fall in eGFR in various late-stage cancer patients (*P* < 0.05). However, the majority of the subjects (*n* = 47, 58.0%) had been previously treated with cisplatin. This may have affected the eGFR baseline which ranged from 40 to 167 ml/min/1.73 m^2^ and acted as a potential source of bias. Khrunin et al. (Khrunin et al., 2010b) defined nephrotoxicity as ≥1 grade of nephrotoxicity of NCI-CTCAE classification, while Tzvetkov et al. used relative change in the eGFR.

In contrast to the aforementioned results, 11 other studies, mostly underpowered, reported no statistically significant association between the *ERCC1* polymorphisms and cisplatin nephrotoxicity (Goekkurt et al., 2009; Chen et al., 2010; KimCurran et al., 2011; Erculj et al., 2012; Khrunin et al., 2012, 2014; Windsor et al., 2012; Khokhrin et al., 2013; Liu et al., 2014; Hattinger et al., 2016; Powrozek et al., 2016). Thus, further studies to disclose molecular mechanisms of ERCC1-mediated cisplatin nephrotoxicity are needed as a scientific basis for a future clinical study.

### Genetic polymorphisms in ERCC2 (XPD)

Significant associations between variants in *ERCC2* and cisplatin-induced nephrotoxicity were reported in four studies (Goekkurt et al., 2009; Kim et al., 2012; Windsor et al., 2012; Powrozek et al., 2016). However, there was high heterogeneity in study characteristics. Two studies (Windsor et al., 2012; Powrozek et al., 2016) were retrospective cohort studies while one was a prospective cohort (Goekkurt et al., 2009) and one was a case-control study (Kim et al., 2012). Three studies focused on individuals of European ancestry (Goekkurt et al., 2009; Windsor et al., 2012; Powrozek et al., 2016) while one study focused on East Asian patients (Kim et al., 2012). Two studies clearly defined nephrotoxicity based on NCI-CTCAE criteria, but there were differences in the nephroxicity threshold [≥grade 2 vs. ≥grade 1; (Windsor et al., 2012; Powrozek et al., 2016)] while two studies did not mention their definitions (Goekkurt et al., 2009; Kim et al., 2012). Of the six SNPs that showed statistically significant associations, only rs13181 survived replication.

Powrozek et al. showed that the A allele of 2251A > C (rs13181; p.Lys751Gln), a missense variant which potentially changes XPD protein expression and modulates nucleotide excision repair (Benhamou and Sarasin, 2002), was associated with a 14 and 4-fold greater cisplatin nephrotoxicity after the second and fourth chemotherapy cycle, respectively, through an allelic (not genotype) association analysis (Powrozek et al., 2016). Another study (Goekkurt et al., 2009) found that ~22% of 133 patients carrying the variant genotype of the *ERCC2* rs13181 and *ERCC2* rs1799793 suffered from grade 2–4 nephrotoxicity, which was significantly higher than in those carrying other genotypes; the OR for nephrotoxicity was 2.27 for the *ERCC2* Asn312/751Gln (rs179979/rs13181, both are missense mutations) haplotype [*P* = 0.005; (Goekkurt et al., 2009)]. In contrast, Windsor et al. (Windsor et al., 2012) found that the AA genotype of *ERCC2* rs13181 had a marginally lower nephrotoxicity risk after the second chemotherapy cycle (OR = 0.23, *p* = 0.044). Patients carrying AA genotype also experienced lower drops in eGFR than the AC/CC genotype (4 vs. 23 mL/min/1.73 m^2^, *P* = 0.021). Five studies displayed contradictory results when compared with the previously mentioned articles (Khrunin et al., 2010b, 2012, 2014; Erculj et al., 2012; Hattinger et al., 2016). Since ERCC2's role in cisplatin nephrotoxicity has not yet been fully understood, studies confirming the role of XPD proteins could help to uncover the molecular mechanisms underlying cisplatin nephrotoxicity. By manipulating ERCC2 gene expressions in suitable renal cell models, the role of XPD proteins in cisplatin nephrotoxicity could potentially be confirmed.

### Genetic polymorphism in SLC22A2 (OCT2)

Genes that encode for drug transport proteins, such as *SLC22A2* (encoding the OCT2 protein) efficiently mediate the cellular uptake leading to high cisplatin accumulation particularly in renal proximal tubule in cells (Miller et al., 2010; Ciarimboli, 2012; Wensing and Ciarimboli, 2013). This condition accelerates the cytotoxic potential of the drug, including nuclear and mitochondrial DNA damage and production of reactive oxygen species (ROS), involved in pathways of apoptosis and necrosis (Miller et al., 2010). A nonsynonymous, missense mutation in *SLC22A2* rs316019 (p.270Ala > Ser; G > T) was studied in seven studies in which four studies concluded that the variant genotype was protective against cisplatin nephrotoxicity.

Filipski et al. (Filipski et al., 2009) and Iwata et al. (Iwata et al., 2012) reported that patients carrying the wild-type genotype of rs316019 (GG) were more susceptible to cisplatin-induced nephrotoxicity compared to the other genotype, as defined by a statistically significantly increased serum creatinine (*P* < 0.05). Iwata et al. reported that wild-type GG had higher increase of serum creatinine than patients carrying the variant GT [1.11 ± 0.37 vs. 0.92 ± 0.15 mg/dL; *P* = 0.04; (Iwata et al., 2012)]. Zhang et al. (Zhang and Zhou, 2012) reported similar results in which the patients carrying the wildtype GG had higher levels of cystatin C than patients carrying the variant genotypes of GT and TT (0.043 ± 0.107 vs. −0.013 ± 0.120 mmol/L; *P* = 0.009). Each of those prospective cohort studies focused on malignant solid cancers in either European Caucasians or East Asians populations. The newest study by Chang et al. (Chang et al., 2017) in American Caucasians patients showed that patients carrying the GG genotype of rs316019 exhibited higher urinary fold changes in kidney injury molecule-1 (KIM-1) at day 3 after cisplatin administration (1.77 × 10^171^; *P* < 0.05) compared to the carriers of the other genotype. Higher reduction in eGFR and increase of blood urea nitrogen (BUN) in rs316019 wild-type GG genotype was also observed in two studies, although this was not statistically significant (Tzvetkov et al., 2011; Hinai et al., 2013). In contrast to these four studies, the remaining three studies (Khrunin et al., 2010a; Tzvetkov et al., 2011; Hinai et al., 2013) did not find any associations, but were generally underpowered, involved multiple types of cancer, and/or included both early and late stages in European and East Asian predominant populations, which may have contributed to the differences in results.

## Discussion

### Summary of main results

To our knowledge, this is the first systematic review conducted to evaluate genetic markers associated with cisplatin nephrotoxicity. We report that eight germline polymorphisms had significant associations with cisplatin nephrotoxicity, of which variants in the genes *ERCC1, ERCC2* (DNA repair) and *SLC22A2* (drug transport) had consistent results across at least two independent populations.

Polymorphic variations of genes associated with the uptake of cisplatin from the renal proximal tubular cells, such as *SLC31A1* (CTR1), *SLC31A, SLC22A1-3* (OCT transport proteins), *ATP7A* and *ATP7B* (Copper-Transporting ATPases 1 and 2), and those that regulate the urinary cisplatin efflux from these cells, such as *SLC47A1* (MATE1), *ABCB1* (MDR1); *ABCG2* (BCRP); *ABCC1-2* (MRP1-2) have been evaluated (Aleksunes et al., 2008; Harrach and Ciarimboli, 2015) but only *SLCC2A2* (SNP rs316019) was associated with cisplatin toxicity. By applying a dominant genetic model, three studies suggested that genotypes GT/TT of *SLC22A2* rs316019 reported significant changes in kidney function biomarker that were considered clinically relevant. Studies from Japan and China reported up to 38% of changes in SCr [Mean difference: −0.19 mg/dL; (Iwata et al., 2012)] and up to 41% of changes in cystatin C [Mean difference: 0.056 mmol/L; (Zhang and Zhou, 2012)] compared to normal values [SCr: 0.6–1.2 mg/dl for male, 0.5–1.1 mg/dl for female; cystatin C: 0.068–0.118 mmol/L regardless gender; (Hosten, 1990; Finney et al., 2000)]. As *SLC22A2* encodes the organic cation transporter 2 (OCT2), the association between rs316019 and SCr and eGFR might be due to an effect on tubular creatinine secretion; however, no clear evidence was found for a relationship between the SNP and cystatin C (Reznichenko et al., 2013). In addition, an elevation in KIM-1 (1.77 × 10^171^ fold) also indicates possible clinical relevance of this SNP (Chang et al., 2017). Further research measuring the eGFR changes and classifying the subjects into widely accepted CTCAE category would provide more insight into the importance of this SNP.

Rs316019 (c.808G > T; p.270Ala > Ser), the only common coding polymorphism within *SLCC2A2* with an allele frequency ranging from 9 to 16%, is reported to cause changes in transporter function (Zolk, 2012). A report by Filipski et al. suggested that T allele in rs316019 is associated with decreased expression of *SLC22A2* in a panel of human cell lines (Filipski et al., 2009). However, polymorphic variants of genes that regulate such transporters (e.g., OCT2, MATE1) in the cell still require further study. This will provide insight into the potential contribution of suspected transporter genes polymorphisms in cisplatin-induced nephrotoxicity, ototoxicity and neurotoxicity.

In cisplatin-induced nephrotoxicity, alteration of DNA repair mechanisms might play a role particularly in renal cells. Nucleotide excision repair genes are involved in the elimination of lesions that lead to distortion of the DNA helix structure (Bowden, 2014) and have been implicated in cisplatin outcome (*ERCC1*), sensitivity and resistance [*ERCC2*; (Bowden, 2014)]. Variations in *ERCC* may affect the repair function through alterations of protein or mRNA expression levels (Xiong et al., 2017) and *Ercc1* mutant mice are deficient in several DNA repair processes that cause accelerated aging, particularly in non- or slowly proliferating organs (Niedernhofer et al., 2006). *ERCC1* rs11615 is a synonymous variant while rs3212986 is located at the 3′ UTR (non-coding region); hence both SNPs are unlikely to produce amino acid changes that affect the DNA repair mechanism. However, the tissue expression quantitative trait loci (eQTL) analysis from GTEx reported a significant association between rs11615, rs3212986, and gene expression in various tissues (Group, 2017). Unfortunately, no association has been found between these SNPs and *ERCC1* expression in kidney cortex tissue. In contrast, *ERCC2* rs13181 (c.2251A > C), a missense non-synonymous variant resulting in amino acid changes from lysine to glycine (p.751Lys > Gln) is more likely to alter the DNA repair capacity (Duell et al., 2000; Lunn et al., 2000). However, the gene involvement in cisplatin-induced nephrotoxicity pathway have not been extensively studied (Zhu et al., 2015). Small study sizes, different ethnicities, varied and mixed cancer types, different combinations of chemotherapy, and different outcome definitions plague most of these analyses. Therefore, a large study with renal function as the primary outcome and with a clinically relevant nephrotoxicity threshold (e.g., ≥grade 2 CTCAE classification) should be conducted, particularly one that will consider a genetic risk score across multiple germline genes, possibly through a comprehensive GWAS or whole exome or whole genome sequencing approach.

### Quality and inconsistency among studies

Most studies did not report study power, provide a sample size calculation or report genotyping quality control tests, raising the possibility of underpowered false negative results [type II error; (Krzywinski and Altman, 2013)] and contributing to inconsistent results (Biau et al., 2008). Other reasons for conflicting results related to series of heterogeneous confounding variables typical in the setting of observational studies of toxicity: (1) differences in definition and grading of nephrotoxicity, (2) differences in baseline patient characteristics that could act as confounders of kidney function, (3) differences in chemotherapy regimens and use of other supportive drugs that could independently or additively predispose to nephrotoxicity (e.g., diuretics), (4) differences in cumulative or density of cisplatin dose and exposure time, (5) differences in the amount and the schedule of hydration across studies, and (6) possibility of gene-gene interactions that affect the cisplatin nephrotoxicity pathway. An example of these inconsistencies are the seven studies that evaluated the *SLC22A2* gene. These studies varied in sample sizes between 53 and 206 patients, used five different nephrotoxicity standards, involved patients across various types of cancer and applied a mix of various different chemotherapy regimens. Moreover, two studies were conducted in women only. Despite these quality reporting issues, we were still able to identify four positively replicated SNPs across three genes. *ERCC1, ERCC2*, and *SLC22A2* warrant further investigation.

### Research and clinical implications

Studies that scan the entire genome, such as GWAS, are needed urgently (Zhu and Zhao, 2007), and can lead to the identification of novel genetic variants associated with nephrotoxicity. But these hypothesis-free approaches do require large sample sizes or rigorously phenotyped populations. The first observational study through genome-wide association and whole-genome sequencing studies to investigate drug-induced kidney disease—the DIRECT study is currently ongoing but the results are still pending (Awdishu et al., 2016). Because of patient heterogeneity, any genetic associations must take clinico-epidemiologic and demographic variations into account [e.g., performance status, regular use of NSAIDs, hypoalbuminemia, cardiac disease; (Kidera et al., 2014; Liu et al., 2014; Bhat et al., 2015; Miyoshi et al., 2016; Sato et al., 2016)], through proper documentation, prospective data collection, and appropriate adjustments during the statistical genetic analyses. Alternatively, population pharmacokinetic and/or pharmacodynamic modeling can be used as the basis of additional pharmacogenetic evaluations.

Despite more abundant reporting on *ERCC1* and *ERCC2* involvement in cisplatin nephrotoxicity, the *SLC22A2* gene appeared to have the most consistent evidence of association with nephrotoxicity (same genetic models and across three different biomarkers (serum creatinine, cystatin C, KIM-1), though none of the studies reported eGFR and a magnitude of effect (i.e., odds ratio). Some studies reported the variants of *ERCC1* and *ERCC2* as risk factors while others reported the same variants as protective factors. In addition, SNP *ERCC1* rs11615 and rs3212986 were not associated with changes in protein and mRNA expression (Woelfelschneider et al., 2008; Gao et al., 2011; Zhuo et al., 2018), and the studies on *ERCC1* and *ERCC2* polymorphisms primarily investigated efficacy as the main outcome, with nephrotoxicity as a secondary endpoint. In contrast, polymorphisms in *SLC22A2*–known to be highly expressed in kidney, have been consistently reported as protective factors against nephrotoxicity. Overall, *SLC22A2* is the most promising candidate gene in predicting cisplatin nephrotoxicity with regard to having a biological explanation related to molecular mechanisms on cisplatin nephrotoxicity, where its encoded protein, OCT2, is directly involved in renal cisplatin uptake (Ciarimboli et al., 2005; Filipski et al., 2009; Yonezawa and Inui, 2011; Yonezawa, 2012). If validated further*, SLC22A2* genetic testing may one day be implemented in the clinical setting, especially variant genotypes of *SLC22A2* rs316019 provide protective factors toward cisplatin nephrotoxicity which may allow higher cisplatin doses to be administered. On the other hand, administration of an OCT2 inhibitor might minimize nephrotoxicity risk (Sprowl et al., 2013; Panesso et al., 2014; Ikemura et al., 2017) but with comparable hematotoxicity (Ikemura et al., 2017) in patients carrying the highest risk genotypes.

In patients at highest risk of nephrotoxicity, a number of potential other therapeutic options are available: magnesium supplementation (Crona et al., 2017) may reduce cisplatin accumulation by regulating the expression of the renal transporters rOCT2 and rMATE1 (Saito et al., 2017); switching to carboplatin may reduce the nephrotoxic potential, even though carboplatin may have lower response rates than cisplatin, along with a range of toxicities different from cisplatin (de Castria et al., 2013).

The systematic approach based on predetermined comprehensive PICOS criteria using established electronic databases is a strength of this review. In addition, we conducted the review according to the PRISMA guideline to minimize the selection bias (Supplementary Table 5). However, we were unable to perform a quantitative comparison and meta-analysis due to heterogeneity in study design, treatments, outcome measures, statistical measurement and the use of effect size.

To summarize, an increasing number of pharmacogenomics studies of cisplatin-induced nephrotoxicity has been published within the past decade. Review of these studies highlighted several genes that potentially affect the risk of cisplatin nephrotoxicity although limitations in study design, lack of reproducible results and lack of studies with sufficient quality remain a concern. In addition to transporter genes, DNA repair genes deserve further investigation to discern their putative role in cisplatin nephrotoxicity. The upcoming results of a genome-wide approach, such as used in the DIRECT study may have an advantage to address the limitations of the current studies, and take one step further toward the application of personalized and precision medicine in cancer patients treated with cisplatin.

## Author contributions

ZZ, SV, and A-HM-vdZ research idea and study design. ZZ and SV search strategy and literature search. ZZ and SV data analysis/interpretation. ZZ and ES assessment of study quality and risk of bias. A-HM-vdZ, RM, and SV supervision or mentorship. ZZ made the first draft of manuscript. A-HM-vdZ, SV, RM, BC, GL, JV, and PB critically reviewed and revised the article. Each author contributed important intellectual content during manuscript drafting or revision and accepts accountability for the overall work by ensuring that questions pertaining to the accuracy or integrity of any portion of the work are appropriately investigated and resolved.

### Conflict of interest statement

The authors declare that the research was conducted in the absence of any commercial or financial relationships that could be construed as a potential conflict of interest.
