# Supplementary material for: Genetic Variations and Cisplatin Nephrotoxicity: A Systematic Review
Source: Front Pharmacol. 2018 Sep 27;9:1111. doi: 10.3389/fphar.2018.01111 (PMC6171472; doi:10.3389/fphar.2018.01111)
Supplement: Supplementary file 1 [file Table_1.DOCX]

Supplementary Material

Genetic variations and cisplatin nephrotoxicity: a systematic review

**Zulfan Zazuli, Susanne Vijverberg, Elise Slob, Geoffrey Liu, Bruce Carleton, Joris Veltman, Paul Baas, Rosalinde Masereeuw, Anke-Hilse Maitland-van der Zee***

**Correspondence:** Anke-Hilse Maitland-van der Zee: a.h.maitland@amc.uva.nl

**Supplementary Table 1.** MeSH terms used in search strategy

| ↓ OR; →AND | Exposure | Determinant | Outcome |
| --- | --- | --- | --- |
| MeSH terms | “Cisplatin”[Mesh Terms] | “Polymorphism, Genetic”[Mesh Terms] | “toxicity”[MeSH Subheading] |
|  |  | “Pharmacogenetics”[Mesh Terms] | “adverse effects”[MeSH Subheading] |
|  |  |  | “Acute Kidney Injury”[Mesh Terms] |
| Free-text terms | “CDDP”[Title/Abstract] | “pharmacogenomics”[Title/Abstract] | “nephrotoxicity” [Title/Abstract] |
|  | “platinum-based chemotherapy”[Title/Abstract] | “SNP”[Title/Abstract] | “renal toxicity” [Title/Abstract] |
