# Supplementary material for: Genetic Variations and Cisplatin Nephrotoxicity: A Systematic Review
Source: Front Pharmacol. 2018 Sep 27;9:1111. doi: 10.3389/fphar.2018.01111 (PMC6171472; doi:10.3389/fphar.2018.01111)
Supplement: Supplementary file 2 [file Table_2.docx]

Supplementary Material

Genetic variations and cisplatin nephrotoxicity: a systematic review

**Zulfan Zazuli, Susanne Vijverberg, Elise Slob, Geoffrey Liu, Bruce Carleton, Joris Veltman, Paul Baas, Rosalinde Masereeuw, Anke-Hilse Maitland-van der Zee***

**Correspondence:** Anke-Hilse Maitland-van der Zee: a.h.maitland@amc.uva.nl

**Supplementary Table 2.** PICOS Framework of the Systematic Review

| Population | Subjects of all age with primary inclusion criteria are subjects who diagnosed with solid tumor cancer, received cisplatin-based chemotherapy, no prior chemotherapy or radiotherapy, do not have preexisted or a history of renal disease. |
| --- | --- |
| Intervention/ Exposure | Genetic polymorphisms found in subjects |
| Comparison | Subjects that have wild-type genetic variations. |
| Outcomes | The nephrotoxicity experienced by the subjects described as categorical and/or continuous variable. If possible, the odds ratio of having nephrotoxicity will be included and analyze in the reviews. |
| Study design | The type of studies included in the reviews will be all toxicity studies determining the association between genetic polymorphism and nephrotoxicity induced by cisplatin-based chemotherapy, regardless the method of genotyping. |
