# Supplementary material for: Genetic Variations and Cisplatin Nephrotoxicity: A Systematic Review
Source: Front Pharmacol. 2018 Sep 27;9:1111. doi: 10.3389/fphar.2018.01111 (PMC6171472; doi:10.3389/fphar.2018.01111)
Supplement: Supplementary file 3 [file Table_3.DOCX]

Supplementary Material

Genetic variations and cisplatin nephrotoxicity: a systematic review

**Zulfan Zazuli, Susanne Vijverberg, Elise Slob, Geoffrey Liu, Bruce Carleton, Joris Veltman, Paul Baas, Rosalinde Masereeuw, Anke-Hilse Maitland-van der Zee***

**Correspondence:** Anke-Hilse Maitland-van der Zee: a.h.maitland@amc.uva.nl

**Supplementary Table 3.** Quality Assessment Form

Each item could get 0 or 1

| **No.** | **Quality aspect** | **Grade** |
| --- | --- | --- |
| 1 | Quality of clinical information | |
|  | 1. 1 point: mention the type of cisplatin-based chemotherapy regimens, the dosage and cycles) |  |
|  | 1. 1 point: adequate [description of] selection of participants, for example, inclusion criteria, nephrotoxicity criteria or objective lab parameters and baseline characteristics) |  |
| 2 | Quality of genotyping | |
|  | 1. 1 point: consideration of the Hardy–Weinberg equilibrium |  |
|  | 1. 1 point: consideration of genotyping quality, for example, by reporting percentage of successful genotyping attempts or cross validation with a different technique) |  |
| 3 | Quality in reporting of study population origin | |
|  | 1. 1 point: mention geographical point of sample collection |  |
|  | 1. 1 point: stratification or exclusion based on ethnicity, or statistical correction for population origin) |  |
| 4 | Quality in terms of sample size and statistical correction for multiple testing | |
|  | 1. 1 point: describe the power analysis to determined sample size |  |
|  | 1. 1 point: 1 point: any correction for multiple testing). |  |
| 5 | Quality of study setup and analysis | |
|  | 1. 1 point: the study included participants not using cisplatin-based chemotherapy (e.g., carboplatin-based or other chemotherapy) |  |
|  | 1. 1 point: the risk of bias was analyzed and statistical interaction term cisplatin-based chemotherapy*genotype was calculated and interpreted correctly, [i.e., subject to the same p-value threshold as main effects]). |  |

The scoring system resulted in an overall quality score of 0-10.
