# Supplementary material for: Genetic Variations and Cisplatin Nephrotoxicity: A Systematic Review
Source: Front Pharmacol. 2018 Sep 27;9:1111. doi: 10.3389/fphar.2018.01111 (PMC6171472; doi:10.3389/fphar.2018.01111)
Supplement: Supplementary file 4 [file Table_4.DOCX]

Supplementary Material

Genetic variations and cisplatin nephrotoxicity: a systematic review

**Zulfan Zazuli, Susanne Vijverberg, Elise Slob, Geoffrey Liu, Bruce Carleton, Joris Veltman, Paul Baas, Rosalinde Masereeuw, Anke-Hilse Maitland-van der Zee***

**Correspondence:** Anke-Hilse Maitland-van der Zee: a.h.maitland@amc.uva.nl

**Supplementary Table 4.** Quality assessment result for selected articles

| **Study** | **Quality of clinical information** | | **Quality of genotyping** | | **Quality in reporting of study population origin** | | **Quality in terms of sample size and statistical correction for multiple testing** | | **Quality of study setup and analysis** | |
| --- | --- | --- | --- | --- | --- | --- | --- | --- | --- | --- |
|  | (a) | (b) | (a) | (b) | (a) | (b) | (a) | (b) | (a) | (b) |
| Xu X, 2012 | 1 | 1 | 1 | 1 | 1 | 1 | 0 | 1 | 0 | 0 |
| Khrunin AV, 2014 | 1 | 0 | 0 | 1 | 1 | 1 | 0 | 0 | 0 | 1 |
| Khrunin AV, 2010 | 1 | 1 | 0 | 0 | 1 | 0 | 0 | 1 | 0 | 0 |
| Tzvetkov, 2011 | 1 | 0 | 1 | 1 | 1 | 1 | 1 | 1 | 0 | 1 |
| Kim SH, 2012 | 0 | 1 | 1 | 1 | 1 | 0 | 0 | 0 | 1 | 1 |
| Windsor RE, 2012 | 1 | 1 | 1 | 0 | 0 | 0 | 0 | 0 | 0 | 1 |
| Khrunin AV, 2012 | 1 | 1 | 0 | 0 | 0 | 1 | 0 | 0 | 0 | 1 |
| Zhang, 2012 | 1 | 1 | 1 | 1 | 1 | 0 | 1 | 0 | 1 | 0 |
| Iwata, 2012 | 1 | 1 | 0 | 0 | 1 | 0 | 0 | 0 | 0 | 0 |
| Filipski, 2009 | 1 | 0 | 0 | 0 | 1 | 1 | 0 | 0 | 0 | 1 |
| Goekkurt, 2009 | 1 | 1 | 1 | 1 | 1 | 0 | 0 | 0 | 1 | 1 |
| Powrozek T, 2015 | 0 | 1 | 1 | 0 | 1 | 1 | 0 | 0 | 0 | 0 |
| Hattinger CM, 2016 | 1 | 1 | 1 | 0 | 1 | 1 | 0 | 0 | 0 | 0 |
| Chen S, 2010 | 1 | 0 | 1 | 0 | 1 | 0 | 0 | 0 | 0 | 1 |
| Erculj, 2011 | 0 | 1 | 1 | 0 | 1 | 1 | 0 | 0 | 1 | 1 |
| Hinai Y, 2013 | 1 | 1 | 1 | 0 | 0 | 0 | 1 | 0 | 0 | 0 |
| Khokrin DV, 2013 | 1 | 1 | 0 | 0 | 1 | 1 | 0 | 0 | 0 | 0 |
| Khrunin AV, 2010 | 1 | 1 | 0 | 0 | 1 | 1 | 0 | 0 | 0 | 1 |
| KimCurran V, 2011 | 1 | 1 | 1 | 1 | 1 | 1 | 0 | 1 | 1 | 0 |
| Lamba JK, 2014 | 1 | 1 | 1 | 1 | 0 | 0 | 0 | 1 | 0 | 1 |
| Liu HE, 2014 | 1 | 1 | 1 | 1 | 1 | 0 | 0 | 0 | 0 | 1 |
| Sprowl JA, 2012 | 0 | 1 | 0 | 0 | 0 | 1 | 1 | 0 | 0 | 0 |
| van der Schoot GGF, 2016 | 1 | 0 | 1 | 0 | 1 | 0 | 0 | 0 | 0 | 1 |
| Wang Z, 2008 | 1 | 1 | 0 | 1 | 1 | 1 | 0 | 0 | 0 | 0 |
| Xu X, 2012 | 1 | 1 | 1 | 0 | 1 | 0 | 0 | 1 | 0 | 1 |
| Yuan ZJ, 2015 | 1 | 1 | 1 | 0 | 1 | 1 | 0 | 0 | 0 | 0 |
| Zhang J, 2012 | 1 | 1 | 1 | 0 | 1 | 1 | 0 | 0 | 0 | 1 |
| Chang C, 2017 | 1 | 1 | 1 | 0 | 1 | 1 | 0 | 0 | 0 | 1 |
